# Disruptions of sexually transmitted and blood borne infections testing services during the COVID-19 pandemic: accounts of service providers in Ontario, Canada

**DOI:** 10.1186/s12913-023-09028-z

**Published:** 2023-01-13

**Authors:** Heeho Ryu, Ezra Blaque, Mackenzie Stewart, Praney Anand, Oralia Gómez-Ramírez, Kinnon R. MacKinnon, Catherine Worthington, Mark Gilbert, Daniel Grace

**Affiliations:** 1 Dalla Lana School of Public Health, University of Toronto, 155 College Street, 5th Floor, Room 556, Toronto, ON M5T 3M7 Canada; 2 Alliance for South Asian AIDS Prevention, Toronto, ON Canada; 3 BC Centre for Disease Control, Vancouver, BC Canada; 4 School of Population and Public Health, University of British Columbia, Vancouver, BC Canada; 5 Canadian HIV Trials Network, Vancouver, BC Canada; 6 School of Social Work, York University, Toronto, ON Canada; 7 School of Public Health and Social Policy, University of Victoria, Victoria, BC Canada

**Keywords:** COVID-19, Sexual health, STBBI testing, Virtual health, Self-sampling, Community-based research, Service providers, Canada

## Abstract

**Background:**

Since the onset of the COVID-19 pandemic in March 2020 in Canada, the availability of sexual health services including sexually transmitted and blood-borne infection (STBBI) testing has been negatively impacted in the province of Ontario due to their designation as “non-essential” health services. As a result, many individuals wanting to access sexual healthcare continued to have unmet sexual health needs throughout the pandemic. In response to this, sexual health service providers have adopted alternative models of testing, such as virtual interventions and self-sampling/testing. Our objective was to investigate service providers’ experiences of disruptions to STBBI testing during the COVID-19 pandemic in Ontario, Canada, and their acceptability of alternative testing services.

**Methods:**

Between October 2020-February 2021, we conducted semi-structured virtual focus groups (3) and in-depth interviews (11) with a diverse group of sexual health service providers (*n* = 18) including frontline workers, public health workers, sexual health nurses, physicians, and sexual health educators across Ontario. As part of a larger community-based research study, data collection and analysis were led by three Peer Researchers and a Community Advisory Board was consulted throughout the research process. Transcripts were transcribed verbatim and analysed with NVivo software following grounded theory.

**Results:**

Service providers identified the reallocation of public health resources and staff toward COVID-19 management, and closures, reduced hours, and lower in-person capacities at sexual health clinics as the causes for a sharp decline in access to sexual health testing services. Virtual and self-sampling interventions for STBBI testing were adopted to increase service capacity while reducing risks of COVID-19 transmission. Participants suggested that alternative models of testing were more convenient, accessible, safe, comfortable, cost-effective, and less onerous compared to traditional clinic-based models, and that they helped fill the gaps in testing caused by the pandemic.

**Conclusions:**

Acceptability of virtual and self-sampling interventions for STBBI testing was high among service providers, and their lived experiences of implementing such services demonstrated their feasibility in the context of Ontario. There is a need to approach sexual health services as an essential part of healthcare and to sustain sexual health services that meet the needs of diverse individuals.

**Supplementary Information:**

The online version contains supplementary material available at 10.1186/s12913-023-09028-z.

## Background

In March 2020, the Government of Ontario, Canada, declared a provincial state of emergency and imposed several measures to contain and manage the spread of COVID-19 [1]. Public spaces, businesses, and gathering places deemed “non-essential” were forced to close and social gathering limits were put in place under the emergency orders [1, 2]. Around this time, the Ontario Ministry of Health and Long-term Care also ordered all “non-essential health services” to be paused [3]. Most sexual health clinics in Ontario were forced to close as a result, and the few that remained open had to reduce their operational hours, or only offer appointments to those who had already been diagnosed with sexually transmitted and blood-borne infections (STBBI) or were experiencing symptoms of STBBI [4, 5].

Many individuals living in Canada and the United States with sexual health needs were unable to access STBBI testing and other sexual health services due to their lack of availability during the COVID-19 pandemic [6–8]. For example, among clients of a provincial sexual health clinic or an online-based STBBI testing service in British Columbia, 66% of respondents with sexual health needs reported that they delayed accessing sexual health services during the COVID-19 pandemic [6]. The closure of their usual place for accessing sexual healthcare was one of the most cited reasons for delaying access to sexual health services [6]. Lack of access to sexual healthcare such as STBBI testing is concerning given that early detection and treatment are essential for the prevention of further transmission, especially HIV [9–11], and that rates of infection for other STBBI such as syphilis have increased by 124% in Canada between 2016 and 2020 [12]. And although there was a 22% decrease in the reported rates of infection for HIV between 2019 and 2020 in Canada, and a general decrease in the reported rates of infection for other STBBI such as chlamydia (29%), gonorrhea (19%), and syphilis (5%) in Ontario during the same period, their precise rates of infection may not be entirely known for some time [13, 14]. The downward trends in the rates of STBBI infection between 2019 and the first year of the COVID-19 pandemic may be due its impact on the availability and access to sexual health testing services and the STBBI surveillance capacities of health authorities, as well as the delays in reporting sexual health data [13–15]. The lack of access to sexual healthcare is especially concerning as it may adversely impact members of sexual and gender minority groups such as gay, bisexual and other men who have sex with men (GBM) and transgender people, as well as racialized minorities, Indigenous peoples, sex workers, and injection drug users who are disproportionately affected by STBBI in Canada [11].

In response to these unmet testing needs, sexual health clinics and service providers have adopted alternative models of care, such as virtual sexual health and self-sampling/testing interventions to enable access to sexual health services for their clients [8, 15–17]. Virtual sexual health and self-sampling/testing interventions have also been shown to be highly acceptable among clients of sexual health services during the pandemic [6, 7, 16, 18]. In Canada, a number of alternative sexual health interventions were available prior to the COVID-19 pandemic, such as online-based STBBI testing services [19, 20], self-testing kits [20], and private e-clinics offering sexual health testing services [21]. In Ontario, the self-testing kits and private e-clinics have not been integrated into the public health system but are instead available for a fee, providing differential access to sexual health testing services based on an individual’s ability to pay for private health services [21]. During this time, there were also efforts underway to make HIV self-testing available across Canada, and in November 2020 the blood-based INSTI HIV self-test was approved for public use by Health Canada [22]. Currently there are on-going trials in Ontario studying the acceptability and uptake of HIV self-testing kits and preliminary results indicate high acceptability and effectiveness of this intervention among sexual health service users [18, 23].

Within this context, the objectives of our study were to: 1) investigate the impact of the COVID-19 pandemic on the availability of STBBI testing services in Ontario through service providers’ experiences of disruptions to STBBI testing; 2) examine how services providers adopted alternative models of sexual healthcare such as virtual services and self-sampling interventions during the pandemic; and 3) assess the perceived challenges and benefits of such interventions among service providers.

### Major developments during the COVID-19 pandemic in Ontario

**Fig. 1:**
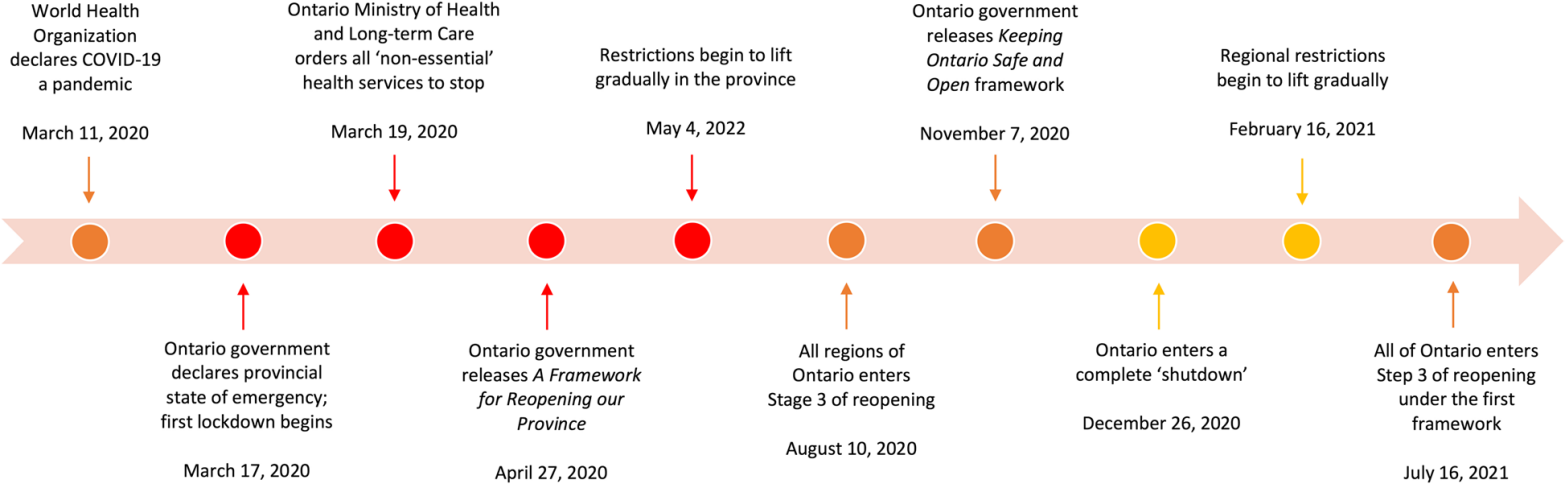
Timeline of major developments during the COVID-19 pandemic in Ontario

Alternative sexual health interventions were an important part of providing care to sexual health clients throughout Ontario during the pandemic as levels of closures and reopenings varied across time and provincial regions. Figure 1 below illustrates a visual timeline of changing COVID-19 measures in Ontario; the first “lockdown” period is represented in red, and the second “shutdown” period is represented in yellow. The first lockdown period began in March 2020 when the Government of Ontario declared a provincial state of emergency, placing social gathering limits and forcing the closure of public spaces, businesses, and gathering places deemed non-essential [1, 2]. All non-essential health services were also ceased under the guidance of the Ontario Ministry of Health and Long-term Care, including sexual health services such as STBBI testing [3–5].

In April 2020, the Ontario government released *A Framework for Reopening our Province* which outlined a gradual three-step approach to easing restrictions, with Step Three involving the reopening of all businesses and workplaces along with relaxed social and public gathering limits [24]. The lockdown that began in mid-March slowly came to an end as the provincial government began to gradually ease the various restrictions, including for non-essential health services [25]. By August 2020, all provincial regions entered Stage 3 of the reopening [25]. Yet with another surge of cases beginning in the fall, the three-step approach was replaced with the *Keeping Ontario Safe and Open Framework* which recategorized public health units into 5 levels [26]. This new approach allowed the provincial government to manage the rising cases of infection by placing varying levels of restrictions on specific public health regions without having to resort to another province-wide lockdown. Despite such efforts, all of Ontario entered a complete shutdown by the end of 2020 [27]. For much of the province, this period of shutdown measures ended in February 2021, but regional lockdowns, shutdowns, emergency breaks, and stay-at-home orders continued to be in place for specific public health regions based on their local rates of infection and COVID-19 health risks [2, 25]. Ontario entered Step Three of the original framework in July 2021, reopening the entire province once again [28].

## Methods

**Fig. 2:**
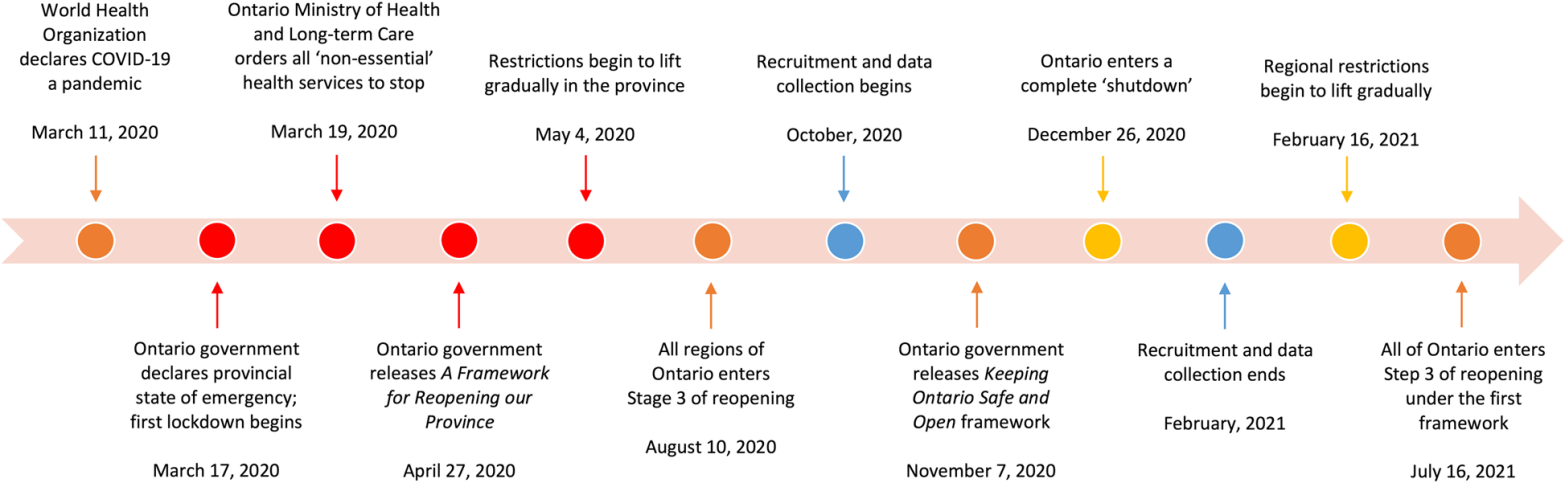
Timeline of data collection in the context of the COVID-19 pandemic in Ontario

Between October 2020 and February 2021, we conducted 60–90 min semi-structured focus groups and interviews with 18 STBBI testing and other sexual health service providers based in Ontario, Canada [29]. Figure 2 below illustrates a visual timeline of our data collection period within the major developments during the COVID-19 pandemic in Ontario; marked in blue, our data collection began after the initial lockdown had been lifted, and ended during the following shutdown period. This analysis is part of a larger community-based research study examining the acceptability of online-based STBBI testing service called GetCheckedOnline (GCO) among GBM and sexual health service providers in Ontario [30, 31]. Recruitment, data collection, and analysis were conducted by three Peer Researchers [HR, MS, EB] self-identifying as members of the GBM community with the support of the Research Coordinator [PA] and Principal Investigator [DG]. A Community Advisory Board comprised of public health workers, therapists, ASO workers, and GBM from across Ontario was consulted throughout the research process to review and direct the course of research. This study was approved by the University of Toronto Research Ethics Board.

### Recruitment

Sexual health service providers were recruited through purposive and snowball sampling methods following grounded theory [32]. Recruitment posters were distributed through email to AIDS service organizations (ASOs), community-based organizations (CBOs), community health centres, public health units, and other sexual health clinics that serve sexual and gender minorities within the province. Posters were also shared through the social media platform Facebook, and participants were encouraged to share this research opportunity with their colleagues. Interested sexual health service providers completed an eligibility screener on SurveyMonkey and those who met the study criteria were invited to participate. Service providers who did not have experience directly working with sexual health service users and/or were not currently working in Ontario were excluded from the study.

### Interview and focus group instruments

The focus group and interview guide was developed by the Principal Investigator [DG] and Research Coordinator [PA], and reviewed by the Peer Researchers [HR, MS, EB]. In both the focus groups and interviews, participants were asked the same questions regarding the changes they experienced within their work and workplace during the COVID-19 pandemic. We asked: *How have services at your workplace changed during the COVID-19 pandemic? Have you remained open throughout the lockdown? How have you scheduled appointments during the pandemic? What has changed for your clients? Do you/have you been offering telehealth services? What kind (video calls, telephone)? How has telehealth services changed your assessment of STI? How has pre- and post-test counselling changed during the pandemic/lockdown?*

Participants were also asked questions about their interactions with clients and any changes they noticed. We asked: *How have conversations about having sex changed throughout the pandemic? Are clients less likely to disclose sexual encounters/behaviours? Do you note any changes in people’s testing behaviour due to the pandemic*? *Is there more reluctance and/or stigma for getting tested/having sex?* For the full interview guide, see supplemental file 1.

Based on participants’ availability, they were invited to participate in focus groups (comprising of 2–3 service providers) where participants were able to engage with each other’s experiences and opinions in a back-and-forth conversational exchange. Due to scheduling conflicts and service providers’ lack of availability during the COVID-19 pandemic, participants were invited to partake in individual interviews when they were unable to participate in focus groups.

### Data collection

Prior to the focus groups and interviews, participants provided their written and informed consent and completed a sociodemographic survey on Qualtrics. Data collection was conducted online using Microsoft Teams due to health concerns regarding COVID-19 and related government restrictions. Virtual interviews using conferencing software have been identified as a secure and suitable alternative to in-person interviews in qualitative research [33]. With the participants’ consent, Peer Researchers conducted 60–90 min long focus groups and interviews which were audio and video recorded and stored on a secure online drive. Audio recordings were transcribed verbatim and each participant was assigned a pseudonym to protect their identity. Based on the principles of theoretical saturation, data collection was concluded when no new insights or themes related to the study developed from the focus groups and interviews [34].

### Analysis

Transcripts were coded using the NVivo 12 software and qualitative analysis techniques informed on grounded theory [35]. After reviewing and developing codes for each transcript, the Peer Researchers [HR, MS, EB] gathered to discuss common themes and emerging patterns. The codes were then refined and organized into overarching themes and subthemes, and each transcript was recoded using the revised framework. The Research Coordinator [PA] and Principal Investigator [DG] were consulted throughout the stages to further refine and develop the codes and themes. The coding and analysis were informed by a literature review conducted prior to the data collection phase.

## Results

### Characteristics of participants

In total, there were 3 focus groups and 11 individual interviews with a diverse group of sexual health service providers (*n* = 18). Participants’ occupation varied from frontline workers at community-based or HIV/AIDS service organizations (*n* = 7), sexual health nurses (*n* = 4), physicians (*n* = 2), public health workers (*n* = 2), clinic counsellors/HIV testing professionals (*n* = 2), and managers of sexual health nurses (*n* = 1). They served a wide range of regions from the Greater Toronto Area (*n* = 7), Ottawa (*n* = 2), and other large urban areas (> 100,000) (*n* = 8) to rural communities (1,000–29,000) (*n* = 1). Most had over five years of experience (*n* = 9), followed by 1–5 years (*n* = 7), and less than one (*n* = 2). Participants were asked about the primary populations they served and were able to choose more than one option. Their clients were primarily gay, bisexual, queer, and other men who have sex with men (*n* = 15), people who use intravenous drug (*n* = 8), trans/gender non-conforming individuals (*n* = 7), sex workers (*n* = 5), youth (*n* = 2), adults between 18–60 (*n* = 1), and immigrants/newcomers (*n* = 1).

### Emerging themes

Three broad themes related to our participants’ experiences with providing sexual health testing services during the COVID-19 pandemic were identified. First, we examine the impact of the COVID-19 pandemic on disruptions to STBBI testing services provided by Public Health Units in Ontario on the overall sexual health landscape in the province. Second, we describe the novel changes in STBBI testing programs such as virtual models of care and self-sampling interventions. Third, we consider how the novel virtual and self-sampling programs were received by service providers in the province and their perceived benefits and challenges.

#### Disruptions to STBBI testing services

Many of our participants reported that public health units across the province had their capacity for STBBI testing reduced “dramatically” since the beginning of the COVID-19 pandemic and the first lockdown (March–May, 2020) in Ontario. They explained that much of public health’s resources and staff were redeployed for COVID-19 management activities such as COVID testing and contact tracing. A director of a local public health unit shared their experience:

[Ontario Public Health’s] priority during [the COVID-19 pandemic] is COVID management, so a lot of our sexual health team nurses have been redeployed to deal with COVID […] [at] our lowest, it was down to one public health nurse dealing with all the STBBI for our region that come in […] and even now it’s like staffing is half of what they used to be because of all the redeployments […] just the volume of services offered has decreased dramatically within our community. (Individual Interview)

Other participants also shared similar narratives and identified the reduced staffing and resources in sexual health clinics run by public health as the primary cause for the lack of availability of STBBI testing services within the province. Participants illustrated that during the first lockdown period, some sexual health clinics were shut down entirely while others had limited operational hours and in-person capacity, which had greatly reduced the availability of STBBI testing services. A sexual health nurse further explained:

Our health unit […] well we have our clinics open. [We were shut] down for two months [in March 2020] then [we] reopened two of our clinics one day a week. And then now we’re up to four clinics one day a week but that’s still half as many clinics as we used to have. And because of COVID we have to space people out more, so we can’t see as many people, and we’ve got fewer staff in the clinic because of [COVID]; so all of that has decreased our capacity [for STBBI testing]. (Focus Group)

Clinic closures and capacity constraints were reported by our participants to be a common experience in both urban and rural regions of Ontario during the pandemic. In the following focus group exchange, a public health worker from Toronto and a sexual health nurse from a rural region shared similar experiences of how the COVID-19 pandemic affected the availability of sexual health clinics and services in their respective localities since the declaration of a provincial state of emergency and first lockdown measures:

*Public health worker:* Most of the health units [in the Greater Toronto Area] have been dealing with such huge numbers of [COVID-19 cases] – like the health units have completely redeployed to deal with COVID case management and all that […] there used to be I think what, like maybe 8 or 10 sexual health clinics affiliated with the city that you could pop into on certain days of the week, at certain times […] I think there’s still one running. Um, there’s some like partnership clinics that we’re able to continue running, but again at like massively reduced capacity.

*Sexual health nurse:* For me over here, it’s the same thing. [Our sexual health testing] program is almost three-quarters gone, all working COVID […] I was covering [2 municipalities over 50km apart] and then like I’m driving you know, all over the place [to provide testing services] […] and all our clinics are postponed for the time being. (Focus Group)

The drastic decreases in public health resources and staff, closure of sexual health clinics, and consequently the limited availability of STBBI testing services were identified by participants as a serious and pressing health issue in Ontario. Some explained that prior to the COVID-19 pandemic, Public Health Units in Ontario provided about half of all STBBI tests conducted within the province. Hence, they explained that the demand for testing was being redirected to other sexual health service providers such as private labs, family doctors, and other sexual health clinics offered by ASOs, CBOs, and community health centres. A director of a local public health unit explained it like this:

I think the statistic is something like, in non-COVID years, Public Health provides about 50 percent of STBBI testing across Ontario, so when we redeploy away from sexual health, I assume – I presume across Ontario, access to sexual health services has decreased dramatically, and that has been a very difficult choice we’ve made here even ourselves […] So if Public Health can’t carry that load, that will have to go somewhere. (Individual Interview)

Indeed, participants affiliated with various sexual health clinics discussed the need to create additional sexual health testing capacities within the communities they served. Some shared that their organizations increased the amount of STBBI testing they offered, or were in the process of opening new sexual health clinics since the end of the first COVID-19 lockdown period. This change was to meet the demand for sexual health testing services still existing during the pandemic, while at the same time facing the existing challenges with the COVID-19 pandemic such as limited in-person capacity for staff and clients and reduced clinic hours. One sexual health nurse illustrated:

We are like a primary care location for HIV clients, so we also, since COVID, have been taking on a lot of STI testing for our [local] community. Because Public Health is working with a lot of the COVID things, we’ve taken over almost all of the STI testing in our area. (Individual Interview)

Within the context of such challenges, many service providers agreed that public health should continue to prioritize COVID-19 management and that the reallocation of resources and staff was necessary. Yet they also expressed that compromising the availability of publicly funded health services, including sexual health, was harmful to the general population as well. For example, a frontline worker from a local ASO shared:

I understand the whole idea of having to reallocate resources to help fight the pandemic. I think at the expense of sexual health or any other health related issue, I think we do a disservice to the public. And I think we need to make sure that sexual health is also seen as an essential service. (Focus Group)

Echoing these sentiments, some participants also highlighted that the lack of publicly available STBBI testing services during the COVID-19 pandemic may have disproportionately affected clients who face additional barriers to testing. This included clients who may not have a general practitioner or access to public or private health insurance, instead having to rely on the limited number of STBBI testing services that are publicly funded. For example, a public health worker demonstrated:

It’s been unfortunate to see services so scaled back and […] access so compromised for so many people. Because again, if – if you don’t have a health card, you can’t go to [just any] clinic or whatever, you have to go to one of the city-run clinics, and if there’s only one and you’re working, or you can’t get in for two weeks, it’s dicey. (Focus Group)

While our participants recognized the necessity of redirecting resources and staff towards COVID-19 management, they also identified the negative health impacts it had on the overall sexual health testing landscape in Ontario. Participants highlighted the essential role publicly funded sexual health clinics and STBBI testing services provide within their communities, especially for clients who may experience additional barriers to testing. There was also an emphasis on the need to view sexual health as an “essential” part of the prevailing understanding and framework of health.

#### Changes within the sexual health landscape

Within the context of challenges such as limited resources, staff, clinic hours, and in-person capacities, our participants shared that most of their sexual health testing services were adapted to be remote during the pandemic to overcome such challenges. They identified a shift away from in-person visits to virtual risk assessment, intake, and counselling sessions using the phone or internet. Some added that in-person visits were available but limited to clients who were in “urgent” need of testing or treatment, such as those with recent exposure, symptoms, or high-risk profiles. A sexual health educator explained in detail:

Our services have gone to majority virtual, so phone or video appointments for almost everything, in-person appointments booked only after our clinician deems them necessary […] they might not even get tested depending on what the guidelines say […] or they might get a [requisition] to bring to a local lab. They might be encouraged to defer testing, depending on the level of lockdown at the moment. (Individual Interview)

The participant further emphasized that the sexual health risks of their clients continued to be weighed against the COVID-19 risks within their region, which informed the decision on providing in-person services on a case-by-case basis. Some clients may be invited to get tested in person, while others may be referred to get tested at labs or even asked to stay at home until a later date. Some participants also added that their organizations also designed and implemented new alternative STBBI testing initiatives to overcome such challenges and risks posed by the COVID-19 pandemic. Two types of programs our participants described are presented in Table 1 below.

**Table 1:** New STBBI testing programs developed during the COVID-19 pandemic in Ontario

Quick Clinic Model	Virtual Clinic Model
Requisition is pre-filled; clients visit any participating location to pick-up a requisition and self-collect samples on-site	Requisition is filled over the phone and sent to a local lab in Ontario; clients visit the lab of choice for testing
Reserved for asymptomatic clients; clients with symptoms or exposure are referred to in-person services	Reserved for low-risk clients; clients with exposure or other risks are referred to in-person services
Only chlamydia and gonorrhea testing are available; blood-based testing is not available	All types of testing are available, including blood-based testing for STI and HIV

For what we describe as the *Quick Clinic* model, requisitions are pre-filled through a medical directive and clients visit any participating location, such as public health units, women’s shelters, ASOs, CBOs, and community health centres to pick-up the requisition and a self-sampling kit. Clients collect the samples onsite and drop them off during the same visit; only chlamydia and gonorrhea testing were available for this program. In the *Virtual Clinic*, a requisition is filled over the phone by a doctor or a nurse and sent directly to the local lab chosen by their client; all STBBI tests, including blood-draw tests for HIV were available. Both programs were reserved for low-risk or asymptomatic clients, and those with exposure, symptoms, or high-risks were referred to in-person services instead.

Participants identified the COVID-19 pandemic and subsequent disruptions to STBBI testing services to be the catalyst for the approval and implementation of their new testing initiatives. Some shared that key stakeholders and decision-makers were persuaded by the necessity of programs such as the Virtual Clinic during this time and their potential benefits such as lower cost and resource dependence. A sexual health nurse demonstrated:

[The Virtual Clinic is] completely COVID driven. One of the nurses came up with that as an idea and said, ‘hey could we do this?’ and within a couple of weeks we got it up and running which is great […] Public Health could be accused of being kind of a little bit behind the times […] and if COVID hadn’t been happening there’s – I don’t think we would have ever gotten that through, but because of COVID we were able to somehow miraculously get it through. (Focus Group)

Others also shared the sentiment that before the pandemic, new ideas and potential innovations in healthcare services had been explored at lengths but never implemented or fully realized within the province. It was further explained that services such as remote assessment, intake, and referral which are now considered to be standard practices were already possible, but had not been adopted by their organizations due to concerns regarding privacy and efficacy. Participants added that such services may not have been available at this point in time without the necessity for the healthcare system to adapt to the challenges of the COVID-19 pandemic. While disruptions within the sexual health landscape has negatively impacted both clients and service providers in Ontario, participants noted that it may have created future opportunities for the development and implementation of new and innovative STBBI testing interventions.

#### Perceived benefits and challenges

Our participants reported their view that new STBBI testing initiatives were highly acceptable and positively received by both their staff and clients. They shared that their staff found these programs much more efficient, cost-effective, and less onerous compared to the traditional clinic-based testing services. Providers also believed that their clients found these programs much more convenient, accessible, safe, and comfortable than clinic-based testing, especially during the pandemic. A director of a local public health unit explained:

[The Quick Clinic model is] actually, uh, a lot easier on everyone administratively just because there’s less staff required. You don’t need a doctor […] you don’t need nurses there in terms of doing assessments, and doing testing, and handing things out […] how we staff it, it’s pretty much with, uh, a programme assistant, and in some of these other places I think they have […] their medical secretaries kind of do the handing out work [and] the labelling of samples. (Individual Interview)

In another focus group, a sexual health nurse also shared:

[Clients of the Virtual Clinic model have] been pretty happy […] they talk to a doctor on the phone and the doctor orders the [tests], they go to the lab and they get tested, and they’re happy because they got tested whereas we were telling them [earlier that] they either didn’t fit our criteria for coming in or being seen, or we’re booking into like three months or three weeks away from now. (Focus Group)

However, some of our participants also recognized the disadvantages of virtual points of care and emphasized the necessity of traditional clinic-based testing being offered in conjunction to the alternative models of sexual healthcare. For instance, participants discussed the challenges with forging full and meaningful connections with their clients and providing emotional support over the phone or video. They also shared the difficulties some clients face with disclosure during virtual assessment without having adequate privacy at home during the pandemic. A sexual health educator explained:

[With virtual services] it’s meant less opportunities for additional education. It’s meant a lot of clients are facing challenges around having a confidential space to have their appointments in, having time set aside […] so like they might be taking their call on their break from work. They might be – I’ve talked to a bunch of people who are walking outside to take the call because otherwise they would be at home with their parents. (Individual Interview).

Nonetheless, our participants recognized the potential benefits of alternative STBBI testing services and programs beyond the COVID-19 pandemic, and hoped that such virtual and self-sampling interventions would continue to be offered in the province. They advocated for a hybrid system involving both alternative and traditional clinic-based models of testing, and emphasized their belief that providing alternative and clinic-based STBBI testing services concurrently could make sure that the varied testing needs of different clients would be met within the province’s sexual health testing landscape.

## Discussion

Our findings suggest that the availability of publicly funded and community-based STBBI testing services was dramatically reduced across Ontario, Canada, during the COVID-19 pandemic. This was due to challenges such as limited resources and staff, closures, reduced operational hours, and lower in-person capacities at sexual health clinics. As a result, there were major disruptions in sexual healthcare across the province and local service providers struggled to meet their clients’ unmet sexual health needs such as STBBI testing, especially during the first lockdown period (March–May, 2020). Our findings support recent studies from Canada and the United States which also suggest that many clients who sought sexual health services during the COVID-19 pandemic were unable to access them due to their lack of availability [6–8]. Within this context, sexual health service providers emphasized that publicly funded sexual health clinics and STBBI testing services are essential for the health and wellbeing of their clients, and that there was a serious need to increase the availability of sexual health services at this time.

With constraints on on-site, face-to-face services during the COVID-19 pandemic, service providers made the transition toward remote points of care in order to continue providing sexual health services to their clients. Providers reported adopting practices such as virtual risk assessment, intake, and counselling sessions using the phone or internet, and some successfully developed novel alternative testing interventions such as virtual and self-sampling STBBI testing programs.

There is evidence from studies conducted before and during the COVID-19 pandemic suggesting that remote, online-based STBBI testing interventions may be highly acceptable among clients of sexual health services [6, 16, 36, 37]. Dulai et al. (2021) found in their analysis of a national cross-sectional survey of GBM conducted before the pandemic that 78.8% of participants in Ontario were likely to use an online-based STBBI testing service in the future, suggesting the intervention’s potential acceptability within this population [36]. Similarly, among respondents of Gilbert et al.’s (2021) online survey conducted during the pandemic among users of either a provincial sexual health clinic or an online-based STBBI testing service, the likelihood of using virtual health services for sexual healthcare was 80%, suggesting the high acceptability of this intervention among sexual health service users [6]. Previous studies have also demonstrated that online-based sexual health interventions using remote video-conferencing technologies were highly feasible and effective tools for sexual health testing and care, including for consultation, education, result delivery, and linkage to treatment [38–40]. We complement this literature by illustrating the acceptability of remote and virtual sexual health services for STBBI testing among sexual health service providers and that such interventions are feasible within the context of Ontario.

The accounts of service providers also serve to illustrate that self-sampling interventions are acceptable among sexual health service providers and feasible within the sexual health service landscape in Ontario. This adds to the emerging literature which suggest that interest and uptake of new STBBI testing methods such as self-sampling and testing were high among sexual health service users during the pandemic [6, 7, 18, 23]. For instance, early findings (2021) from Ontario demonstrate that GBM in the province find HIV self-testing highly acceptable and show high interest in using the intervention [18]. The authors of the study note the potential for various local clinics, agencies, organizations to coordinate a pick-up program for HIV self-testing kits in the future [18], and our data supports that there is indeed an appetite amongst local service providers to create partnerships with the intent to provide novel STBBI testing services such as self-sampling kits within their communities. Similarly, the results of a pharmacy-based rapid HIV testing trial conducted in Alberta and Newfoundland and Labrador, Canada also found that community-based novel HIV testing programs were acceptable among both clients and service providers and feasible [41]. Prior studies have also demonstrated that self-sampling for sexual health testing using urine and oropharyngeal, vaginal, penile, and anal/rectal swabs is acceptable and feasible among various sexual health service users [38, 42–45]. We thus recommend that the capacity of local agencies to provide such services be strengthened through public funding, allocation of resources, and creating training opportunities during and beyond the COVID-19 pandemic. Increasing the capacity of local agencies to provide more sexual healthcare such as STBBI testing and treatment may also improve the capacity of public health to carry out other critical public health functions as well.

Our service providers believed that alternative models of sexual health testing services such as virtual and self-sampling testing programs were more convenient, accessible, safe, comfortable, cost-effective, and less onerous compared to the traditional clinic-based testing services, and that they filled the gaps in testing caused by the COVID-19 pandemic. At the same time, as other studies have pointed out our participants recognized the challenges with alternative STBBI testing services such as: difficulty forging connections with clients and providing emotional support; challenges some clients may face with accessing virtual services without access to the internet and necessary technology; missed opportunities for further education and resource connection; and challenges with sexual health disclosure without adequate privacy at home [16, 17, 46, 47]. As such, our findings support calls to develop and implement new and innovative models of sexual health service delivery such as virtual testing and self-sampling/testing alongside traditional clinic-based services to expand the options that are available in the sexual health landscape in and beyond the COVID-19 pandemic [6–8, 11]. Offering varied models of sexual health testing concurrently may reduce the potential negative impacts of any one particular model on an individual’s experience with, and access to, sexual health testing services.

Novel STBBI testing initiatives such as online-based testing and HIV self-testing may benefit sexual health service providers and clients generally by increasing access and availability, reducing costs and resources, and improving the overall quality and experience with testing [47]. These new modalities may also be essential in meeting the Pan-Canadian STTBI Framework for Action and UNAIDS 95–95-95 goal by 2030 by helping address issues regarding health equity and equal access to sexual healthcare and education within populations made vulnerable by STBBI [11, 48]. Sexual health issues and STBBI impact groups and individuals differently based on their social determinants of health, including but not limited to, their levels of education and income, employment status, housing status, access to health services, and experience of discrimination based on gender, sexual orientation, and race [49]. For instance, studies have found that groups such as GBM who experience social stigma and discrimination based on their sexual orientation and gender identity have poorer physical, mental, and sexual health outcomes and are much more likely to experience substance use issues [50]. Poor mental health among GBM have been linked to greater likelihood of engaging in high-risk sexual activities and issues with substance use [50, 51], and there is also evidence suggesting that GBM in Canada are disproportionately affected by poor mental health issues such as stress, anxiety, depression, and loneliness both before and during the COVID-19 pandemic [52, 53]. Opportunities to reduce barriers to accessing sexual health and STBBI testing services, such as virtual and self-sampling/testing interventions could therefore greatly benefit individuals and groups who may experience higher levels of vulnerabilities to sexual health concerns and risks, even beyond the COVID-19 pandemic.

Despite the numerous potential benefits of alternative sexual health testing models, MacKinnon et al. (2020) identified legislative and political barriers to the implementation of such STBBI testing services in Ontario prior to the pandemic [54]; they suggest that there is a need to “make a case” for novel STBBI testing models toward provincial stakeholders and decision-makers by highlighting their anticipated positive impacts such as increased rates of sexual health testing and lower costs for testing within the overall public healthcare system [54]. Indeed, our sexual health service providers identified the COVID-19 pandemic and related disruptions to STBBI testing as the catalyst for the approval and implementation of their new testing initiatives, and demonstrated the necessity and success of making a case for alternative models of testing.

At the same time, there is also a need to ensure the equitable distribution of such testing innovations within the province. Within the current sexual health landscape in Ontario for instance, a two-tiered system exists in which individuals can “pay to skip the line” in accessing sexual health testing by purchasing sexual health self-testing kits or paying for private e-clinic visits [20, 21]. Meanwhile, our service providers emphasized the essential role that publicly funded STBBI testing services serve in the province. They further highlighted the significance and importance of publicly funded STBBI testing services for individuals who may experience additional barriers to testing such as those without any status or insurance. Within this context, there is a need for publicly-funded novel STBBI testing interventions which can be accessible regardless of an individual’s socioeconomic status and financial abilities, and can expedite access to testing in a timely manner beyond the COVID-19 pandemic. As service providers in this study emphasized, there is a strong need to view sexual health as an essential part of our understanding and framework of health, and this includes ensuring the equitable distribution of sexual health access and innovations within the province. It is thus crucial for further research in implementation science to explore the sustained and equitable growth of sexual health innovations within the institutional and provincial contexts of the public health landscape in Ontario.

### Limitations

Our study is unique in that it provides important and timely insights into the disruptions of sexual health testing services from the perspective and experiences of service providers during the COVID-19 pandemic. While many studies have explored sexual health service disruptions from the perspective of service users, to our knowledge there is a limited number of research providing a detailed account of service disruptions from the experiences of service providers. Yet our findings are limited in that they rely on the personal accounts and perceptions of sexual health service providers who may hold positive bias related to the effectiveness and acceptability of novel STTBI testing services within their workplace. Further research using implementation science needs to work closely with service providers who have developed new alternative testing interventions to: 1) evaluate quantitative measures of their effectiveness; 2) understand how such services could be improved, expanded, and scaled-up in the province; and 3) explore the ways in which to increase the approval and uptake of alternative sexual health testing interventions among policymakers, decision-makers, and stakeholders. Additionally, our findings may not be generalizable beyond the context of Ontario or the COVID-19 pandemic. At the same time, an important strength of our community-based research study is that Peer Researchers led the research and guided its process together with a Community Advisory Board, enhancing the credibility and validity of our study findings.

## Conclusions

The accounts of sexual health service providers in Ontario suggests that many individuals seeking sexual health services during the COVID-19 pandemic may not have their sexual health needs met due to the lack of available services. New and innovative sexual health services such as virtual sexual health services and self-sampling for STBBI were found to be acceptable among sexual health service providers and feasible within the context of Ontario during the pandemic, and our data demonstrates that alternative sexual health interventions can help alleviate the disruptions in testing caused by the pandemic. Delays in receiving STBBI testing and treatment can be detrimental to the health and well-being of individuals and their communities, especially for those who experience higher vulnerabilities to HIV. Hence, it is of utmost importance that sexual health is understood as an essential part of our overall health, and that sexual health services continue to be provided in an equitable and timely manner during and beyond the COVID-19 pandemic. More research using implementation science is needed to further examine and evaluate the acceptability, feasibility, and effectiveness of virtual and self-sampling/testing interventions within the province. Future research should also explore the ways in which the growth and expansion of sexual health innovations can be sustained and equitable within the shifting political, legislative, and institutional contexts of Ontario’s public health landscape.

## Supplementary Information

**Additional file 1. **

## Data Availability

Data collected for this study is not publicly available due to confidentiality as participants may potentially be identified through the information embedded within the data. Any questions regarding the data can be directed to the corresponding author.

## Abbreviations

STBBI: Sexually transmitted and blood-borne infections
GBM: Gay, bisexual and other men who have sex with men
GCO: GetCheckedOnline
ASO: AIDS service organization
CBO: Community-based organization
